# Silencing SOCS1 via Liposome-Packed siRNA Sustains TLR4-Ligand Adjuvant

**DOI:** 10.3389/fimmu.2019.01279

**Published:** 2019-06-04

**Authors:** Dagmar Hildebrand, Camila Metz-Zumaran, Greta Jaschkowitz, Klaus Heeg

**Affiliations:** ^1^Department of Infectious Diseases, Medical Microbiology and Hygiene, Heidelberg University Hospital, Heidelberg, Germany; ^2^DZIF German Center for Infection Research, Braunschweig, Germany

**Keywords:** vaccination, SOCS1, SOCS1-silenced APC, siRNA, liposomes, TLR4-adjuvant

## Abstract

Infectious diseases remain one of the leading causes of death worldwide. Vaccination is a powerful instrument to avert a variety of those by inducing a pathogen-specific immune response and ensure a long-lasting protection against the respective infection. Nevertheless, due to increasing numbers of immunocompromised patients and emergence of more aggressive pathogens existing vaccination techniques are limited. In our study we investigated a new strategy to strengthen vaccine adjuvant in order to increase immunity against infectious diseases. The strategy is based on an amplification of Toll-like receptor 4 (TLR4) -induced activation of antigen-presenting cells (APCs) by turning off a powerful endogenous inhibitor of APC-activation. TLR4 signaling induces the release of cytokines that bind autocrine and paracrine to receptors, activating the Janus kinase (JAK) 2/signal transducers and activators of transcription (STAT) 3 cascade. Subsequently, STAT3 induces expression of suppressor of cytokine signaling (SOCS) 1 that terminates the inflammatory response. In the approach, TLR4-adjuvant monophosphoryl lipid A (MPLA)-stimulated monocyte-activation is reinforced and sustained by silencing SOCS1 via lipid nanoparticle-enclosed siRNA (L-siRNA). L-siRNA is transported into primary cells without any toxic side effects and protected from early degradation. Through lipid core-embedded functional groups the lipid particle escapes from endosomes and releases the siRNA when translocated into the cytoplasm. SOCS1 is potently silenced, and SOCS1-mediated termination of NFκB signaling is abrogated. Consequently, the MPLA-stimulated activation of APCs, monitored by release of pro-inflammatory cytokines such as IL-6, TNFα, and IL-1β, upregulation of MHC class II molecules and costimulatory CD80/CD86 is strongly enhanced and prolonged. SOCS1-silenced APCs, pulsed with liposomal tetanus light chain toxin (TeTxLC) antigen, activate autologous T cells much more intensively than SOCS1-expressing cells. Importantly, expansion of cocultured CD4^+^ as well as CD8^+^ T cells is remarkably enhanced. Furthermore, our results point toward a broad T helper cell response as TH1 typical as well as TH2 characteristic cytokines are elevated. Taken together, this study in the human system comprises a translational potential to develop more effective vaccines against infectious diseases by inhibition of the endogenous negative-feedback loop in APCs.

## Introduction

Antigen-presenting cells (APCs) are pivotal for the activation of the immune system and the establishment of immunological memory. They are considered as promising targets to enhance immune reactivity in the fight against infectious diseases. APC-based vaccination strategies with *ex vivo* pulsed and activated myeloid cells provide protection against infection with pathogens such as leishmania (1), Herpes simplex virus (2, 3), and Candida albicans (4) in mouse models.

*In vivo*, APCs such as monocytes, macrophages, and Dendritic cells (DCs) recognize pathogen associated molecular patterns (PAMPs) by receptors such as the Toll-like receptors (TLRs) (5). Binding of ligands to extracellular or intracellular TLRs is followed by APC migration to secondary lymphoid organs and presentation of the processed antigens from pathogens to T cells. Importantly, antigens can be presented on MHC class II complex to activate T helper cells as well as MHC class I to mediate CD8 T cell proliferation (6). Additionally, TLR-signaling mediates the expression of cytokines that orchestrate innate as well as adaptive immunity and induces upregulation of costimulatory molecules such as CD80 and CD86 to effectively activate T cell expansion and built a memory.

TLR-ligands have great potential to adjuvant vaccination and are examined for efficiency and safety in many clinical studies (7). The TLR4-ligand monophosphoryl lipid A (MPLA) is well described and has gained FDA approval for use in vaccines such as Cervarix (8). MPLA is a low-toxicity, immunologically active derivative of the lipid A region of lipopolysaccharide (LPS). A common method of MPLA stimulation implies the incorporation into liposomes, artificially constructed vesicles consisting of one or more phospholipid bilayers. During uptake of MPLA-liposomes (L-MPLA) by APCs, MPLA binds and activates membrane-associated TLR4 resulting in MyD88- and TRIF-dependent signaling, subsequent NFκB activation and induction of inflammatory cytokines (9–11). In addition it provokes several additive effects including a depot effect and intracellular processing by both major histocompatibility antigens type 1 and type 2. MPLA forces and directs antigen-specific induction of antibodies and cell-mediated immunity to a variety of antigens (12). Therefore, L-MPLA have been employed as an effective adjuvant system in human trials for different proposed vaccines to, for example, malaria, HIV-1, meningococcal type B disease, and different cancers (12–14).

The aim of adjuvant-mediated APC-activation is finally an increase in antigen-specific T cell expansion and the development of memory cells. Generally, higher peak values of effector T cells correspond to larger amounts of memory T cells. Conventional vaccines, that include whole organisms or large proteins, are highly effective in inducing T cell expansion but include unnecessary antigens that often induce allergenic and/or counterproductive responses (15). Peptide vaccines are an attractive alternative strategy for the induction of highly targeted immune responses, without allergenic and/or reactogenic sequences (16, 17). However, peptide vaccines are mostly poorly immunogenic and often fail to accomplish a sufficient activation and diversity of T cells to receive immunity (18). Therefore, improved adjuvant strategies are needed.

A promising approach to improve peptide vaccination is turning off endogenous negative regulators of APC-activation. During infection-mediated as well as adjuvant-mediated APC activation, negative feedback inhibitors soon terminate the activation. The stimulated release of cytokines and the following cytokine receptor activation lead to activation of the Janus kinase (JAK)2/ signal transducers and activators of transcription (STAT)3 signaling cascade. In consequence, STAT3-dependent inhibitors of cytokine signaling such as the suppressor of cytokine signaling (SOCS)1 are induced to terminate activation (19). SOCS1 is a member of the SOCS and cytokine-induced Src homology 2 (SH2) protein (CIS) family of intracellular proteins and part of the classical negative feedback system. The inhibitor suppresses cytokine receptor signaling by mediating proteasomal degradation of components of the JAK/ STAT signaling to prevent downstream signal transduction (20). Furthermore, it terminates TLR-ligand induced NFκB signaling (21, 22). By controlling cytokine production and antigen presentation, SOCS1 has emerged as regulator of the magnitude of the immune response toward pathogens (23–26). Thus, interfering with SOCS1 should provoke a stronger and prolonged activation of APCs and subsequently result in a more effective adjuvant-induced immune response and protection against infectious diseases.

Recently a mouse study on a vector-based vaccination strategy against HIV provided results regarding a benefit for humoral and cellular response through silencing SOCS1 in APCs (27). However, insertion of plasmids into host DNA is unfeasible in humans because of safety issues.

We investigated whether blocking SOCS1 via a siRNA-mediated approach increases adjuvant-induced activation of primary monocytes and the transduced expansion of antigen-specific T-cells. siRNA-mediated loss of function approaches receive increasing attention in vaccination. However, there are several challenges to persist such as the transport of naked siRNA through the cell membrane, the endosomal escape, and the susceptibility to degradation. To achieve a directed transport of effective amounts of siRNA into the cytoplasm of primary monocytes we made use of a lipid-based low toxicity drug delivery system termed coatsomes (NOF America corporation, White Plains, NY). The coatsomes contain two sensing motifs that can respond to the intracellular environment. Tertiary amines in the core respond to the acidic environment (endosome/lysosome) and mediate a destabilization of the endosomal membrane. This leads to the release of the lipid nanoparticle into the cytoplasm. In the reducing conditions of the cytoplasm the embodied disulfide bond is cleaved and the siRNA is freed. That way, the siRNA is protected from early degradation, escapes endosomes toward the RNA-induced silencing complex (RISC) in the cytoplasm and mediates degradation of target mRNA.

In our approach we strengthen L-MPLA-stimulated activation of primary human monocytes by L-siRNA-mediated silencing of SOCS1, shown by an increased and prolonged expression of pro-inflammatory cytokines and costimulatory molecules. Tetanus light chain toxin (TeTxLC) peptide- pulsed, SOCS1-silenced APCs induced an intense expansion of autologous CD4^+^ and CD8^+^ T cells. The additionally assessed cytokine profile of the cocultures revealed TH1 as well as TH2 cytokines, confirming a broad T cell response.

Taken together, we demonstrate that inhibiting SOCS1 by lipid-nanoparticle packed siRNA is an effective strategy to support TLR4 vaccination adjuvant and increase peptide vaccination immunogenicity.

## Materials and Methods

### Isolation and Culturing of Primary Cells

PBMCs were isolated from fresh blood or buffy coat from healthy donors by density gradient centrifugation (Biocoll separating solution, 1.077 g/ml, Biochrom AG, Berlin, Germany). CD14^+^, CD3^+^, CD4^+^,CD8^+^ cells were magnetically labeled with beads (MiltenyiBiotec) and selected via the autoMACS separator (autoMACS, program: possel, Miltenyi Biotec, Bergisch Gladbach, Germany). Purified cells were cultured in RPMI 1640 (Merck, Darmstadt, Germany) supplemented with 100 IU/ml of penicillin, 100 μg/ml streptomycin and 10% heat inactivated fetal calf serum (Promocell, Heidelberg, Germany) at 37°C in a humidified atmosphere in the presence of 5% CO2.

Monocytes were cultured in 2 ml RPMI/FCS/Pen/Strep in 24 well cell culture plates (Greiner Bio-One, Kremsmünster, Österreich) in a concentration of 1 × 10^6^ cells/ml medium. For monocyte/T cell cocultures 250 000 monocytes were co-cultured with 500,000 CFSE-labeled T cells (from the same donor) per ml in 24 well cell culture plates.

### Monophosphoryl Lipid A (MPLA) Liposomes

Eighteen milligram Phosphatidylcholine, two milligram Phosphatidiylglycerol, and five milligram Cholesterol (Merck, Darmstadt, Germany) were dissolved in 5 ml Chloroform in a flask. Fifty microliter of dissolved MPLA (Avanti Polar Lipids, Inc. Alabaster, Alabama USA) in Chloroform (Carl Roth, Karlsruhe, Germany) (1 mg/ml) was added to the lipid suspension. For empty-liposomes no MPLA was added. Chloroform was then evaporated with a rotatory evaporator (35°C, 600 mBar, rotatory level 2.5, 3 h) until a phospholipid film was left in the flask. For complete evaporation of Chloroform, the film was dried overnight. The next day the film was dissolved in 2 ml PBS. While dissolving, liposomes form spontaneously with inclusion of MPLA molecules. Finally, suspension was filtrated for homogenization and analyzed by Nanoparticle Tracking Analysis (NTA) on a NanoSight NS300 (Malvern Panalytical GmbH, Kassel, Germany) for size distribution and particle concentration.

### Labeling of Liposomes

MPLA-liposomes were fluoresecently labeled with the PKH67 Green Fluorescent Cell Linker Kit for General Cell Membrane Labeling (Merck, Darmstadt, Germany) following the manufacturer's protocol. In brief, 1 ml of liposome suspension was pelleted (3,000 × g in the high-speed centrifuge for 10 min) (Heraeus Biofuge, Marshall scientific, Hampton, USA) and resuspened in 1 ml Diluent C from the Kit. Six microliter PKH67 dye was added, mixed by gentle pipetting and stored for 5 min at RT. By adding 2 ml 10% BSA in PBS spare dye was quenched. Liposome suspension was pelleted, washed two times with 10 ml serum-free RPMI media and finally dissolved in 1 ml serum-free RPMI. For analyzing the uptake of liposomes, primary monocytes were incubated with the PKH67-stained liposomes at a liposome/monocyte ratio of 75:1 for 1, 3, or 18 h at 37°C or 4°C, respectively. After extensive washing the FITC signal was quantified by flow cytometry (FACS Canto, BD Biosciences). Overlays were performed with the Weasel v2.5 software (WEHI, Melbourne, Australia).

### Flow Cytometry

After stimulation as indicated monocytes were analyzed for surface markers with antibody staining (1 h on ice): α-CD80-PE, α-CD86-PE, α-HLA-DR-FITC (BD Biosciences, Heidelberg, Germany). After washing the cells 3 times with PBS mean fluorescence was recorded using the FACS DIVA V 4.12 software on a FACS Canto (BD Biosciences). Overlays were performed with the Weasel v2.5 software (WEHI, Melbourne, Australia).

### Enzyme-Linked Immunosorbent Assay (ELISA)

Commercially available ELISA kits were utilized for detection of human IL-1β, IL-2, IL-4, IL-6, IL-10, IFNγ, TNFα (BD OptEIA ELISA Set; BD Biosciences Pharmingen, Heidelberg, Germany). Assays were performed with cell-free supernatants according to the manufacturer's instructions. Absorbance was measured on a SUNRISE Absorbance reader (Tecan, Salzburg, Austria) and analyzed with Magellan software.

### Western Blotting

2 × 10^6^ cells were harvested and washed with PBS. For whole cell lysates monocytes were lysed in 50 μl RIPA buffer (50 mM Tris-HCl, pH7.4; 1% Igepal; 0.25% sodium deoxycholate; 150 mM NaCl; 1 mM EDTA; 1 mM PMSF; 1 mg/ml each aprotinin, leupeptin and pepstatin; 1 mM Na3VO4; and 1 mM NaF). Samples were vortexed and incubated 30 min on ice. Lysates were then cleared via centrifugation at 14,000 g for 20 min. Equal amounts of cell lysates were used for separation by SDS-PAGE (12.5%). After semi-dry transfer onto nitrocellulose membranes (Whatman Protran nitrocellulose membrane; neoLab, Heidelberg, Germany), the latter were blocked with 5% (w/v) BSA in TBS/0.1% (v/v) Tween-20 for 1 h at RT. Probing was performed with antibodies: Anti-p(y701) STAT3, anti-STAT3, anti-p65, anti-Actin (Cell Signaling Technology, Danvers, MA, USA), and anti-Tetanus Toxin light chain (bioTechne GmbH, Wiesbaden, Germany). Detection was based on enhanced chemiluminescence (ECL; Perkin Elmer, Groningen, Netherlands).

### Quantitative Reverse Transcription PCR (RT-qPCR)

Total RNA was extracted from 2 × 10^6^ cultured primary human monocytes using the high pure RNA isolation kit (Roche, Mannheim, Germany) according to the manufacturer's protocol. RNA preparations were then quantified by spectrophotometry (NanoDrop ND-100 Spectrometer, Peqlab, Erlangen, Germany) and equal amounts were reverse transcribed using Reverse Aid First Strand cDNA synthesis kits (Thermo Fischer Scientific, Karlsruhe, Germany). Obtained cDNA was used for quantitative PCR utilizing the “SYBR green ROX mix” (Thermo Fischer Scientific, Karlsruhe, Germany) and the following sequence-specific primers with regard to expression of protein coding genes: Actin fwd 5′-AGA GCT ACG AGC TGC CTG AC-3′, actin rev 5′- AGC ACT GTG TTG GCG TAC AG-3′, SOCS1 fwd 5′-TCC CCC TCA ACC CCG T-3′, SOCS1 rev 5′-CAT CCG CTC CCT CCA ACC-3′, IL-1β fwd 5′-AGC TGA TGG CCC TAA ACA GA-3′, IL-1β rev 5′-GCA TCT TCC TCA GCT TGT CC-3′.

### siRNA/TeTxLC—Liposomes

Lipid solution (A) of COATSOME® SS-20/3A-P04 (NOF America corporation, White Plains, NY): cholesterol (Merck, Darmstadt, Germany): SUNBRIGHT GM-020 (NOF America corporation, White Plains, NY) = 70:30:3 (mol) was prepared in 90% t-BuOH. siRNA solution (B) was prepared by mixing 100 μl of human SOCS1 siRNA (NM_003745) or contr. siRNA (SIC003) (Sigma-Aldrich, Taufkirchen, Germany) aqueous solution (133 μg/100 μl) and 50 μl 20 mM malate buffer (pH 4.00, 30 mM NaCl). For enclosure of tetanus light chain toxin (List Biological Laboratories, Inc., California, USA) 5 μg/100 μl was added instead of siRNA. While vortexing 400 μl of the lipid solution, siRNA solution (or TeTxLC) was gradually added dropwise and the mixed solution was taken in a 1 ml syringe (C). Then, 225 μl of (C) was injected into 1 ml malate buffer, with vigorous stirring (D). Thereafter, the mixture was further stepwise dialysed with PBS. Adding 2 ml PBS was followed by centrifugation for 1 h at 1,400 rpm in a concentrator plus (Eppendorf, Wiesloch, Germany). Procedure was repeated four times. Final siRNA concentration 66.5 μg/ml. In the experiments 50 μl/ml and 100 μl/ml L-siRNA, 40 μl/ml L- TeTxLC was used.

### T Cell Proliferation Assay

Monocytes were treated as indicated. After 3 days, Carboxyfluorescein succinimidyl ester (CFSE) -labeled T cells (CD3^+^, CD4^+^ or CD8^+^), isolated from the same donor, were added in a ratio of 1:2. For CFSE-labeling T cells were incubated 10 min at r. t. in 0.3 mM CFSE/PBS (MolecularProbes, San Diego,CA, USA) and thereafter intensively washed. For determination of T cell proliferation 250 000 monocytes /ml were co-cultured with 500 000 CFSE-labeled T cells/ml in 24 well cell culture plates (Greiner Bio-One, Kremsmünster, Österreich). After 5–6 days, cell divisions were analyzed by determining the FITC signal using a FACScanto (BD Biosciences, Heidelberg, Germany).

### Statistical Analysis

The comparison of two data groups were analyzed by Mann– Whitney *U* test (one-tailed, confidence intervals 95%) with ^*^p ≤ 0.05, ns, not significant. Additionally, Kruskal-Wallis Test (one-way ANOVA on ranks) was performed. Software: GraphPad Prism Version 5.0.

## Results

### MPLA-Liposomes Activate Primary Monocytes

**Figure 1:**
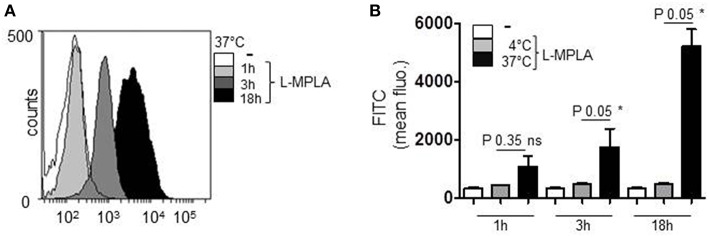
Human primary monocytes were incubated with the PKH67-stained liposome-embedded MPLA (L-MPLA) at a liposome/monocyte ratio of 75:1 for 1, 3, or 18 hours at 37°C. After extensive washing the FITC signal was quantified by flow cytometry. **(A)** Shown is the overlay of histogram produced with WEASEL flow cytometry software. **(B)** Depicted is the quantification of **(A)** and two more experiments. Additionally shown are the results of cells that were incubated with L-MPLA at 4°C. Shown in **(B)**: columns are the mean of three different donors/experiments (*n* = 3) + standard deviation (std) as error bars. Statistics: The comparison of two data groups (line above the bars depicts the compared groups) were analyzed by Mann– Whitney *U* test, one-tailed, confidence intervals 95%, *p ≤ 0.05; ns, not significant. Additionally performed test: Kruskal-Wallis. Number of groups: 9; *P*-value 0.0021; sum value**; the medians vary signif. (*P* < 0.05).

Monophosphoryl Lipid A (MPLA) containing liposomes (L-MPLA), dissolved in chloroform were used as TLR4-adjuvant. MPLA was added to a mixture of Phosphatidylcholine, Phosphatidiylglycerol, and Cholesterol. The mixture was dried in the rotatory evaporator forming a thin lipid film. During resuspension in PBS the L-MPLA form spontaneously. Homogeneous liposome size was achieved by filtration. L-MPLA concentration was determined by Nanoparticle Tracking Analysis (NTA). NTA also showed that the L-MPLA were homogenously distributed in the solution. For evaluation of the mode of uptake of liposomes into primary cells, we stained the lipid core with the green fluorescent dye PKH67. CD14^+^ monocytes were isolated from healthy donors or buffy coat by density gradient centrifugation and antibody-based magnetic cell separation. Afterwards, cells were incubated with PKH67-stained L-MPLA at a liposome/monocyte ratio of 75:1 for one, three or 18 h by incubation at 37°C. After extensive washing the uptake of fluorescent L-MPLA was quantified by flow cytometry, measuring the FITC signal. The histogram overlay in Figure 1A and the associated quantification of further experiments (Figure 1B) shows that liposomes were taken up effectively. The strongest FITC signal was seen after 18 h. Additionally, cells were incubated with L-MPLA at 4°C. At this temperature no endocytosis should occur. As expected, at 4°C the mean fluorescence of cells did not increase, as it was the case in untreated monocytes. This shows that no significant passive uptake or unspecific adherence of the lipid particles occurred (Figure 1B).

**Figure 2:**
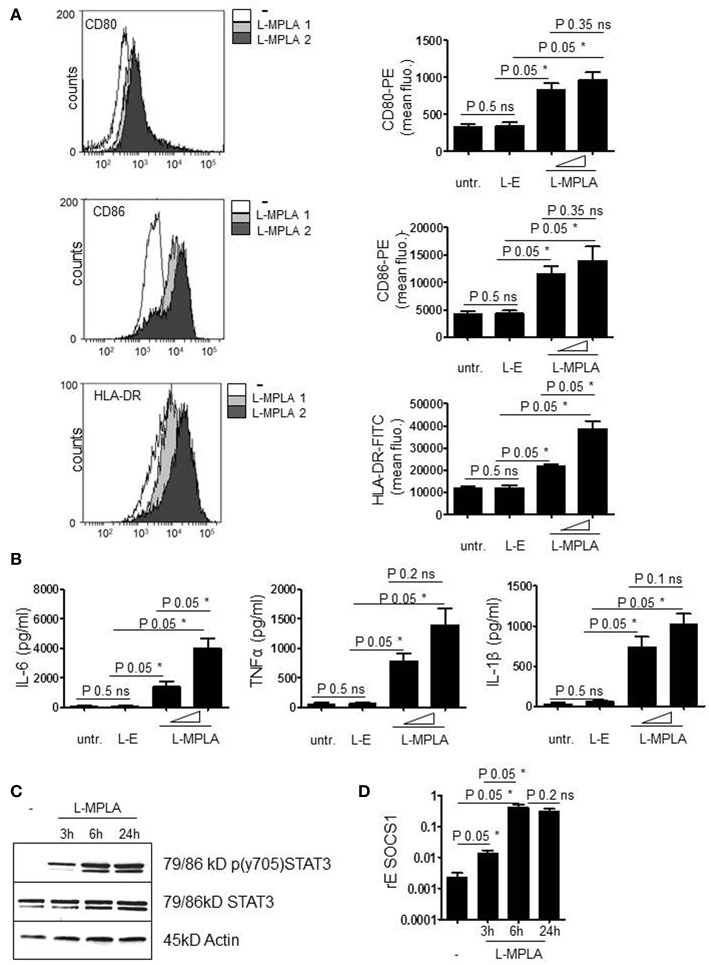
**(A)** Purified cells were incubated with L-MPLA at a liposome/monocyte ratio of 25:1 or 75:1. After overnight incubation, monocytes were analyzed for activation markers (CD80/86) and HLA-DR expression with antibody staining and flow cytometry. Shown are histogram overlays (WEASEL flow cytometry software) (L-MPLA 1 = liposome/monocyte ratio 25:1 L-MPLA 2= 75:1) and the associated quantification of *n* = 3. Quantification includes cells incubated with liposomes generated without MPLA (L-E). Performed test: Kruskal-Wallis. Number of groups: 4; CD80: *P*-value 0.0378; sum value*; CD86: *P*-value 0.0378; sum value*; HLA-DR: *P*-value 0.0249; sum value* the medians vary signif. (*P* < 0.05). **(B)** Supernatant of cultures defined in **(A)** was used for ELISA analysis to detect IL-6, TNFα, and IL-1β. Kruskal-Wallis. Number of groups: 4; IL-6: *P*-value 0.0237; sum value*; TNF: *P*-value 0.030; sum value*; IL-1β: *P*-value 0.0261; sum value* the medians vary signif. (*P* < 0.05). **(C)** Monocytes, incubated with L-MPLA (liposome/monocyte ratio 75:1) for the indicated timepoints were lysed and applied for western blot analyses with anti-p (Tyr701) STAT3, anti-STAT3, and anti-β-Actin (loading control). Shown is one representative blot out of three experiments. **(D)** RNA of cells, treated as described in **(C)**, was isolated and cDNA produced. mRNA induction was analyzed by qRT PCR using sequence specific Primer for SOCS1 and SYBR Green master mix. Results were normalized against housekeeping gene GAPDH. Kruskal-Wallis. Number of groups: 4; SOCS1: *P*-value 0.0216; sum value* the medians vary signif. (*P* < 0.05). A, B, D Columns are the mean of three different donors/experiments (*n* = 3) + standard deviation (std) as error bars. Shown *p*-values: The comparison of two data groups (line above the bars depicts the compared groups) were analyzed by Mann–Whitney *U* test, one-tailed, confidence intervals 95%, *p ≤ 0.05; ns, not significant.

Next, we checked whether endocytosed L-MPLA activated monocytes. Cells were cultivated at a liposome/monocyte ratio of 25:1 and 75:1. After overnight incubation, cells were analyzed for activation markers (CD80/CD86) and MHC2 (HLA-DR) expression by antibody staining and flow cytometry. Figure 2A shows that liposomes without MPLA (“empty liposomes” L-E) had no effect on monocyte activation. Incubation with L-MPLA (liposome/monocyte ratio of 25:1) increased the surface expression of CD80/86 and HLA-DR significantly (*P*-value 0.05). Higher L-MPLA concentration (75:1) further increased expression of the activation markers. Furthermore, we evaluated the expression of inflammatory cytokines of L-MPLA-activated monocytes by ELISA and observed a strong and significant gain in release of IL-6, TNFα, and IL-1β (liposome/monocyte ratio of 25:1: *P*-value 0.05, 75:1 *P*-value 0.05) which was not the case in untreated and L-E -treated cells (Figure 2B).

### L-MPLA Stimulate SOCS1 Induction

Early during APC activation, JAK2/STAT3 signaling is induced due to the release of cytokines and the subsequent cytokine receptor activation. STAT3-induced inhibitors of cytokine signaling such as SOCS1 are induced to terminate the activation status of the cells. The western blot in Figure 2C confirms that L-MPLA treatment stimulated phosphorylation and thereby activation of STAT3. After 3 h, tyrosine phosphorylation of STAT3 was clearly detectable and further increased after 6 h of stimulation. Tyrosine phosphorylation persisted at least up to 24 h (Figure 2C). To further evaluate whether STAT3-mediated and TLR4-mediated NFκB signaling induces SOCS1 expression, quantitative Real-time PCR was performed. Figure 2D shows induction of SOCS1 after 3 h of L-MPLA treatment (*P*-value 0.05). Gene expression peaked after 6 h of stimulation and was high for at least 24 h.

### SiRNA-Mediated Knockdown of SOCS1

Having confirmed that L-MPLA induced SOCS1, which is known to negatively regulate the magnitude of immune response, we aimed to increase monocyte activation by silencing SOCS1. siRNA against SOCS1mRNA (αSOCS1 siRNA) and a control siRNA were packed into lipid particles, termed coatsomes® (NOF America corporation, White Plains, NY). An issue with liposome-packed siRNA is the escape from endosomes toward the RNA-induced silencing complex (RISC) into the cytoplasm. Coatsomes contain two sensing motifs that can respond to the intracellular environment. Tertiary amines respond to the acidic condition in the endosome and destabilize the endosomal membrane which triggers the escape of the coatsome. The reducing conditions in the cytoplasm lead to a cleavage of the disulfide bonds in the coatsomes resulting in siRNA release. Thereby the siRNA is protected from early degradation and is directed to its target mRNA (28, 29).

**Figure 3:**
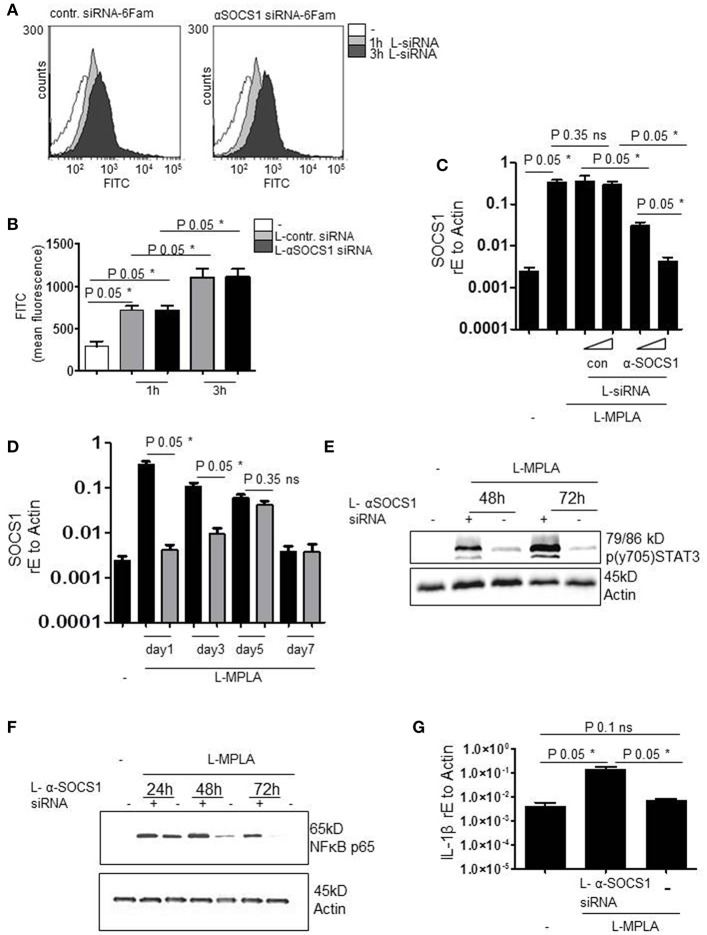
**(A)** Primary human monocytes were incubated with coatsome (NOF Corporation)-packed siRNA-6Fam against SOCS-1 (L-αSOCS siRNA) or an unrelevant sequence (L-con siRNA) for 1h or 3h. Uptake of liposomes was detected by analyzing FITC signal by a FACSCanto. Shown is the overlay of FACS histogram produced with WEASEL flow cytometry software. **(B)** Quantification of **(A)** and two more experiments. Kruskal-Wallis: number of groups: 5; *P*-value 0.0162; sum value* the medians vary signif. (*P* < 0.05). **(C)** L-MPLA-activated cells, treated with increasing concentrations of L-αSOCS siRNA or L-con siRNA (50μl/ml, 100μl/ml L-siRNA) for 24 h, were lysed. RNA was isolated, cDNA produced and used for qRT PCR analyses of SOCS-1. Results were normalized against GAPDH values. Kruskal-Wallis: number of groups: 6; *P*-value 0.0119; sum value* the medians vary signif. (*P* < 0.05). **(D)** Cells were stimulated with MPLA and treated for the indicated timepoints with L-αSOCS siRNA (100μl/ml L-siRNA). RT PCR with SOCS1-specific Primers was performed. Kruskal-Wallis: number of groups: 9; *P*-value 0.0038; sum value** the medians vary signif. (*P* < 0.05). **(E,F)** Cells were cultured for the indicated timepoints with L-MPLA ± L-αSOCS1 siRNA, before lysing. Western blot analyses was performed with **(E)** p (y704) STAT3 **(F)** anti-p56 and anti-Actin (loading control)-specific antibodies. **(G)** Monocytes were treated as described in **(E)** for 72 h, RNA was isolated, cDNA produced and used for rtPCRs with IL-1β specific primers. Data were normalized against Actin. Kruskal-Wallis: number of groups: 3; *P*-value 0.0390; sum value* the medians vary signif. (*P* < 0.05). In **(B,C,F)** is the mean + std, *n* = 3 shown. Statistics: The comparison of two data groups (line above the bars depicts the compared groups) were analyzed by Mann–Whitney *U* test, one-tailed, confidence intervals 95%, *p ≤ 0.05; ns, not significant.

To verify successful packaging of siRNA into lipid particles (L-siRNA) and L-siRNA-mediated transport into primary monocytes, fluorescently labeled 6-FAM (6-Carboxyfluorescein)-siRNA was used. After 1 h and after 3 h of incubating cells with L-siRNA (αSOCS1 siRNA and control siRNA) and repeated washing with PBS, siRNA was detected inside the cells by flow cytometry. Overlays of FACS histograms show that incubation with 6-Fam L-siRNA for 1 and 3 h resulted in a higher FITC signal. This implies the effective delivery of L-siRNA (Figure 3A). The increase of FITC signal was significant for both L-siRNAs and both incubation timepoints (*P*-value 0.05). Comparable amounts of αSOCS1 siRNA and control siRNA were taken up (Figure 3B).

Next, we investigated whether L-αSOCS1 siRNA effectively silences SOCS1. Therefore, we stimulated primary monocytes with MPLA-liposomes (ratio 75:1) and added in parallel increasing amounts of L-αSOCS1 siRNA or L-con siRNA. After 24 h, we evaluated SOCS1 mRNA levels by rtPCR. We observed a clear reduction of L-MPLA-induced SOCS1mRNA level down to basal levels at higher siRNA concentrations. L-con siRNA exerted no effect on SOCS1 mRNA level which excluded any unspecific influence of the coatsomes (Figure 3C). Then we checked how long the knockdown is stable. Figure 3D shows that L-MPLA-stimulated SOCS1 is significantly (*P*-value 0.05) downregulated for 3 days. After 5 days, single treatment-induced knockdown is not stable any more. The L-MPLA-stimulated induction of SOCS1 decreased from day 1 to day 5 and was gone after seven days of culture (Figure 3D).

### Silencing SOCS1 Extends L-MPLA-Induced APC Activation

To verify that the observed decrease in SOCS1 mRNA has an effect on SOCS1 targets, we measured STAT3 tyrosine phosphorylation. SOCS1 targets JAK2 for degradation and thereby stops STAT3 phosphorylation. Figure 3D shows that SOCS-1-silenced cells contained much more pSTAT3 than SOCS-1-expressing cells. This was the case after 48 h and after 72 h of L-MPLA stimulation. After 72 h pSTAT3 seemed to accumulate in the cell (Figure 3E). Additionally, abundance of NFκB subunit p65 was assessed by western blot analyses. P65 is known to be a target of SOCS1 in terms of ubiquitination and degradation (21, 22). Figure 3F shows that L-MPLA- induced p65 decreased after 24 h and was hardly detectable after 72 h. The knockdown of SOCS1 correlated with a prolonged high p65 level which was still high after 72 h. As a consequence of the sustained NFκB signaling, L-MPLA-stimulated IL-1β gene induction was significantly (*P*-value 0.05) higher in SOCS-1-silenced cells after 72 h of L-MPLA stimulation (Figure 3G).

**Figure 4:**
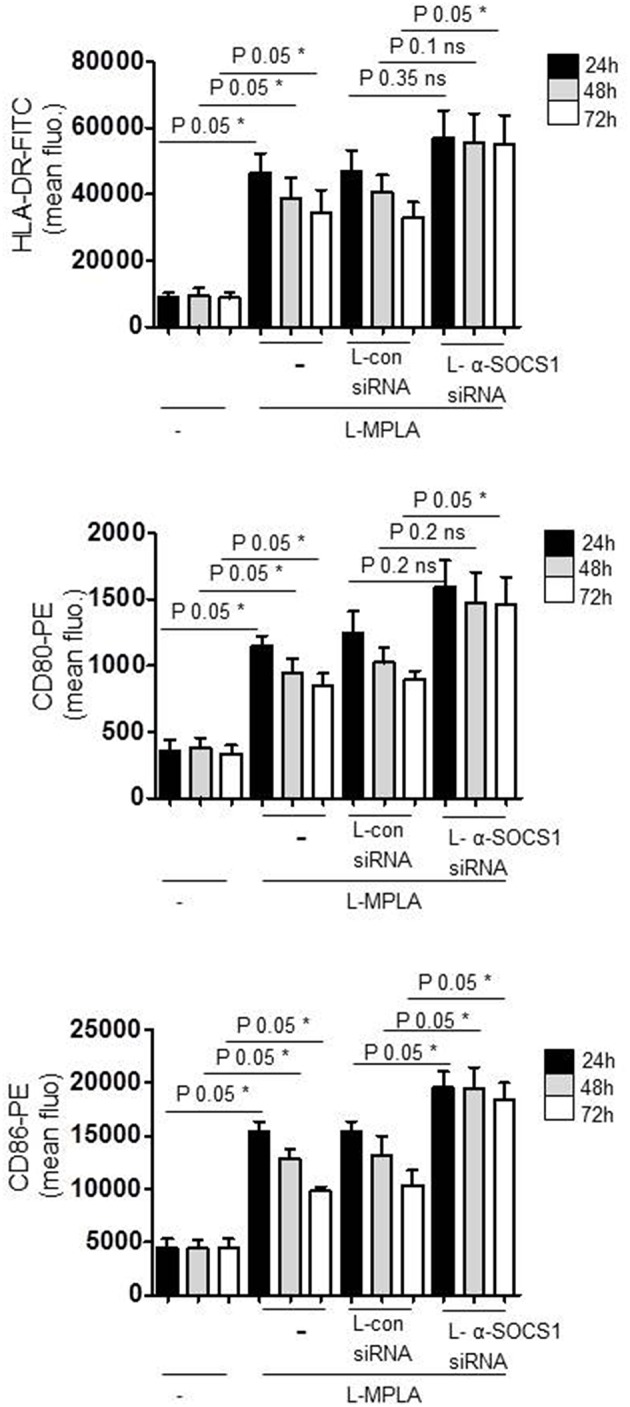
Cells were cultured with liposome-embedded MPLA (L-MPLA) alone or in combination with liposome-packed control siRNA (L-con siRNA) or L- αSOCS1siRNA for the indicated time points and analyzed for surface expression of CD80, CD86, and HLA-DR with specific antibodies (CD80-PE, CD86-PE, and HLA-DR-FITC) on a FACSCanto. Columns: mean of three different donors/experiments (*n* = 3) + standard deviation (std) as error bars. Statistics: Mann– Whitney U test, one-tailed, confidence intervals 95%, *p ≤ 0.05; ns, not significant. Line above the bars depicts the compared groups. Additionally performed test: Kruskal-Wallis. Number of groups: 12; HLA-DR: *P*-value 0.0056; sum value**; CD80: *P*-value 0.0027; sum value**; CD86: *P*-value 0.0009; sum value***. The medians of all three vary signif. (*P* < 0.05).

For APC-stimulated T cell activation, the duration of sent signals is critical to reach a sufficient proliferation threshold for lymphocyte-mediated immunity. For this reason we measured the duration of APC-activation in SOCS1-silenced cells and in SOCS1-expressing cells. Antibody staining and flow cytometry was performed. Figure 4 shows that the surface expression of L-MPLA-stimulated CD80/CD86 and MHC2 (HLA-DR) decreased stepwise within 72 h of observation. In contrast, costimulatory factors and MHC2 were stable increased for 72 h on αSOCS1 siRNA-treated cells. After 72 h the level of CD80, CD86, and MHC2 on SOCS1-silenced cells was significantly higher (*P*-value 0.05) than in cells treated with control siRNA (Figure 4).

### Amplification of Antigen-Specific T Cell Activation

**Figure 5:**
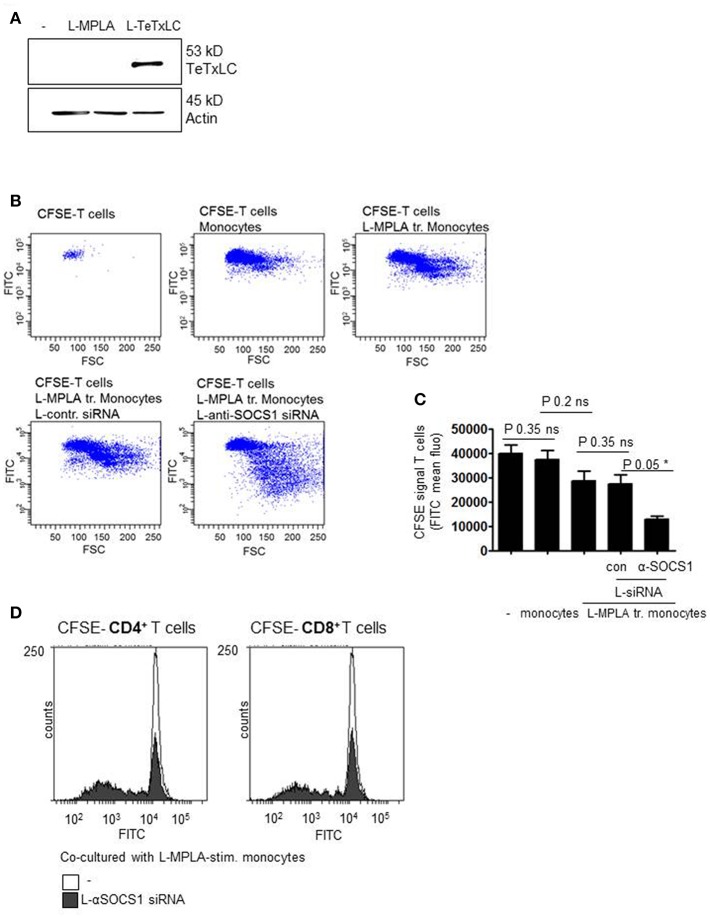
**(A)** Monocytes were stimulated with L-MPLA and treated with liposome-packed Tetanus light chain toxin (L-TeTxLC). The next day lysates for western blot analyses were produced and blottet with anti-TeTxLC-specific and anti-actin- antibodies. **(B)** L-MPLA-activated, TeTxLC-loaded monocytes with or without L-αSOCS1-siRNA were co-cultured with CFSE-labeled CD3^+^ T cells from the same donor. After 5–6 days T cell proliferation was determined by flow cytometry (FITC signal). Shown are dot blots and **(C)** Quantifications of **(B)** and three more experiments. Shown is the mean + std, *n* = 4. Statistics: Mann– Whitney *U* test, one-tailed, confidence intervals 95%, *p ≤ 0.05; ns, not significant. Line above the bars depicts the compared groups. Additionally performed test: Kruskal-Wallis. Number of groups: 5; *P*-value 0.0729; sum value: not significant; the medians do not vary signif. (*P* < 0.05). **(D)** CFSE labeled CD4^+^ or CD8^+^ T cells were co-cultured with L-MPLA ± L- αSOCS1 siRNA for 6 days and analyzed at a FACSCanto for fading FITC signal. Shown are the overlays of histogram produced with WEASEL flow cytometry software.

Then we introduced tetanus light chain toxin (TeTxLC) as vaccination antigen in our system. To obtain presentation of TeTxLC antigen on monocytes, recombinant TeTxLC was shuttled into primary monocytes via liposomes (L-TeTxLC). To verify the uptake of the peptide, western blot analyses with cell lysates with anti-TeTxLC specific antibody was performed. Figure 5A shows that TeTxLC was detected in the cell lysates, which confirms the transport of the antigen into primary monocytes.

In order to check whether the knockdown of SOCS1 increases TeTxLC- specific T cell activation, T cell proliferation assays were performed. Therefore, L-MPLA-activated and L-TeTxLC-pulsed monocytes with or without αSOCS1-siRNA were co-cultured with CD3^+^ T cells. As all donors received tetanus vaccination previously, we assume a TeTx specific priming of T cells. To assess lymphocyte activation, T cells were labeled with Carboxyfluorescein succinimidyl ester (CFSE). CFSE is used to quantify proliferation due to the progressive halving of fluorescence following each cell division. After five-six days of co-culture T cell proliferation was determined by flow cytometry. The dot blots and associated quantifications (Figures 5B,C) show that L-MPLA-activated monocytes induce proliferation of lymphocytes. The treatment with control-siRNA had no additional effect. The siRNA-mediated knockdown of SOCS1 in monocytes strongly increased the proliferation of CD3^+^ T cells. Further experiments verified that the enhanced T cell proliferation corresponds to both CD4^+^ and CD8^+^ T cells (Figure 5D). The flow cytometry histogram overlays in Figure 5D show that after 6 days CD4^+^ as well as CD8^+^ cells cocultured with L-MPLA stimulated, SOCS1-silenced monocytes proliferated much better (decrease in CFSE signal) than those cultured with SOCS1 expressing cells.

**Figure 6:**
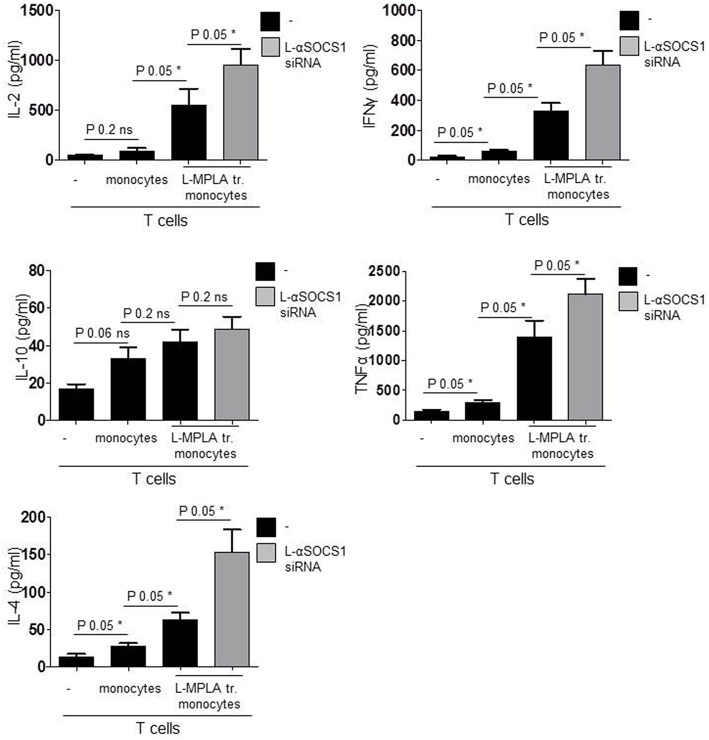
Shown are IL-2, INFγ, TNFα, IL-10, IL-4 ELISA results of supernatant collected from co-cultures: CD3 T cells with untreated monocytes, L-MPLA-stimulated cells or L-MPLA+ L- αSOCS1 siRNA (gray bars). Shown is the mean + std (error bars), *n* = 3. Statistics: The presented comparison of two data groups were analyzed by Mann– Whitney U test, one-tailed, confidence intervals 95%, *p ≤ 0.05; ns, not significant. Additionally performed test: Kruskal-Wallis. Number of groups: 4; IL-2: *P*-value 0.0261; sum value*; INFγ: *P*-value 0.0156; sum value*; IL-10: *P*-value 0.0501; sum value: ns. TNF: *P*-value 0.0188; sum value*; IL-4: *P*-value 0.0156; sum value: *The medians of all measured cytokines, except IL-10, vary signif. (*P* < 0.05).

Finally we measured cytokines in the supernatant of co-cultures. After six days, we quantified IFNγ and TNFα (TH1 response), IL4 (TH2), IL-10 (Tregs), and IL-2. As shown in Figure 6, co-cultures with SOCS1-silenced monocytes contained significantly (*P*-value 0.05) more IL-2, IFNγ, TNFα, and IL-4 than cultures with SOCS1-expressing cells. IL-10 release was also increased although not significantly (*P*-value 0.2). Thereby we conclude that the combination of L-MPLA-activation with abrogation of SOCS1 is sufficient to increase a broad APC-mediated antigen-specific T cell response.

## Discussion

The aim of our study was to increase the TLR4-adjuvant-mediated APC activation and APC-sent inflammatory signals to achieve a stronger T cell response. Since cytokines are considered important effector molecules for many different adjuvants, including TLR-ligands (30), we intended to break inhibition of SOCS1-mediated cytokine receptor signaling and TLR signaling. The negative feedback inhibitor SOCS1 targets Janus kinases (JAKs) of the JAK/STAT cascade and NFκB components to control genes encoding mediators of inflammation (20–22). Thereby, SOCS1 critically regulates the magnitude of inflammatory response.

Hence, inhibiting SOCS1 seems to be a promising target to increase vaccination efficiency. A mouse study on a small peptide antagonist of SOCS1 showed promising results. The palmitic acid-coupled peptide that corresponds to the activation loop of JAK2 (pJAK2 1001-1013) protected mice against lethal vaccinia and encephalomyocarditis virus infection (31). Furthermore, the same peptide was shown to enhance antigen-presenting capacity of human DCs and tumor cell antigen-specific cytotoxic T cell responses (32). However, as pJAK2 is not the only target of SOCS1, direct inhibition of its protein expression might be a more potent approach.

Song et al. could demonstrate in a mouse study that inhibition of SOCS1 in APCs attenuates antigen presentation to control HIV-1-specific humoral and cellular responses. In their approach SOCS1 was silenced in bone marrow-derived DCs via transduced lentiviral siRNA vectors (LV-SOCS1-siRNA). LV-SOCS1-siRNA-DCs immunized and TLR ligand (LPS, PolyI:C, R837) challenged mice enhanced HIV Envelope-specific cytotoxic T lymphocyte and antibody responses. Furthermore, they show that the immune response toward HIV DNA vaccine followed by stimulation with TLR ligand can be enhanced by coimmunization with SOCS1-siRNA plasmid DNA (33). Nevertheless, in humans vaccination with viral and plasmid vectors is still striking. The possibility of insertional mutagenesis and delivery of antibiotic resistance that is usually inserted into the expression plasmid as selection marker implies a safety risk. Furthermore, the lack of stability of injected vectors, possible lethal immunogenic responses to viral vectors and minor transfection efficiency are issues that need to be solved.

In our study we tested an alternative liposome-based approach that might be more applicable for vaccination of humans. Liposomes and lipid nanoparticles have become important carrier systems in vaccine development and the interest for liposome-based vaccines has markedly increased. Several advantages such as low toxicity and a high plasticity make liposomes attractive for vaccination approaches. The composition of the lipid particles allows the entrapping of soluble antigens such as peptides and nucleic acids within the aqueous inner space of liposomes as well as intercalation of lipophilic compounds into the lipid bilayer. In RNA-mediated intervention of protein expression liposomes provide effective delivery of the nucleic acid that is highly susceptible to degradation. However, endosomal escape toward the RNA-Induced Silencing Complex (RISC) in cytoplasm for successful degradation of target mRNA is a problem. Here, we used a lipid-based low toxicity drug delivery system termed coatsomes® (NOF America corporation). The coatsomes contain two sensing motifs. After receptor-mediated uptake, the intact lipid core escapes from the endosomes due to rupture of the endosomal membrane (proton sponge effect). In the reducing conditions of the cytoplasm the embodied disulfide bond is cleaved and the siRNA is able to contact the RISC complex and mediate degradation of its target. Via this technique SOCS1 is effectively silenced in the L-MPLA activated monocytes for three days and downregulation of SOCS1 target proteins is prevented. In consequence, L-MPLA-stimulated activation of monocytes is increased and elongated. The increased activation leads to a pronounced gain of antigen-specific T cell proliferation, which is essential for the development of immunity. Importantly, the L-siRNA-mediated knockdown of SOCS1 induced by a single treatment is stable for three days and abrogated after five days. The L-MPLA induced expression of SOCS1 decreases from day one to day seven. At day seven SOCS1 expression level was as low as in untreated cells. Thus, long-lasting adverse side effects are unlikely but should be taken into consideration.

Besides CD4^+^ T cells also CD8^+^ T cells were more activated when co-cultured with SOCS1-silenced monocytes. This implies that the liposome-packed vaccination antigen TeTxLC is delivered into the cytosol which is a critical step for presentation on MHC class I and the sequential elevation of antigen-specific CD8^+^ T cell responses.

It must be mentioned that beside upregulation of inflammatory signals, silencing SOCS1 might also sustain expression of inhibitory factors that are STAT3-dependend. STAT3 is known to be a key regulator of immuno-suppressive factors such as Programmed cell death 1 ligand 1(PD-L1) (34). PD-L1 is known to inhibit T cell proliferation and induce Tregs (35, 36). Nevertheless, we observed that SOCS1-silenced monocytes stimulate a stronger T cell proliferation than SOCS1-expressing cells. The cytokine profile points toward a TH1 response not toward Tregs. However, additional application of anti-PD-L1 or anti PD-1 antibodies might be beneficial for an increase in T cell activation.

In summary, siRNA-mediated silencing of SOCS1 increases TLR4-adjuvant-stimulated monocyte activation and enhances monocyte-mediated T cell responses. As the vaccination peptide in our system can be easily exchanged the liposome-strategy could be applied against many different extracellular and intracellular pathogens including tumorviruses. Thereby the approach could be especially useful for vaccination against infectious diseases mediated such as leishmaniosis, malaria, or HIV. In addition this approach could also strengthen vaccination efficacy against oncogenic viruses (e.g. HPV).

Taken together, our study provides useful information for the development of a new vaccination strategy in humans against infectious diseases.

## Ethics Statement

The study was carried out in accordance with the recommendations of the ethics committee of the Medizinische Fakultät Heidelberg with written informed consent from all subjects. All subjects gave written informed consent in accordance with the Declaration of Helsinki. The study (taking of blood samples from healthy donors and treatment of blood leukocytes with microbial stimuli) was reviewed and approved by the ethics committee of Medizinische Fakultät Heidelberg.

## Author Contributions

KH and DH designed the study with essential input from CM-Z and GJ. DH wrote the final manuscript. DH, CM-Z, and GJ performed the experiments. All authors read the manuscript and discussed the results.

### Conflict of Interest Statement

The authors declare that the research was conducted in the absence of any commercial or financial relationships that could be construed as a potential conflict of interest.
